# Supplementary material for: Association between serum 25-hydroxyvitamin D levels and Early Vascular Aging in young and middle-aged adults
Source: PLoS One. 2026 May 20;21(5):e0349409. doi: 10.1371/journal.pone.0349409 (PMC13189320; doi:10.1371/journal.pone.0349409)
Supplement: S2 File — (DOCX) [file pone.0349409.s002.docx]

Supplementary figures and table

## Supplementary figures

**S1 Fig. Flowchart of the participants enrollment process.**


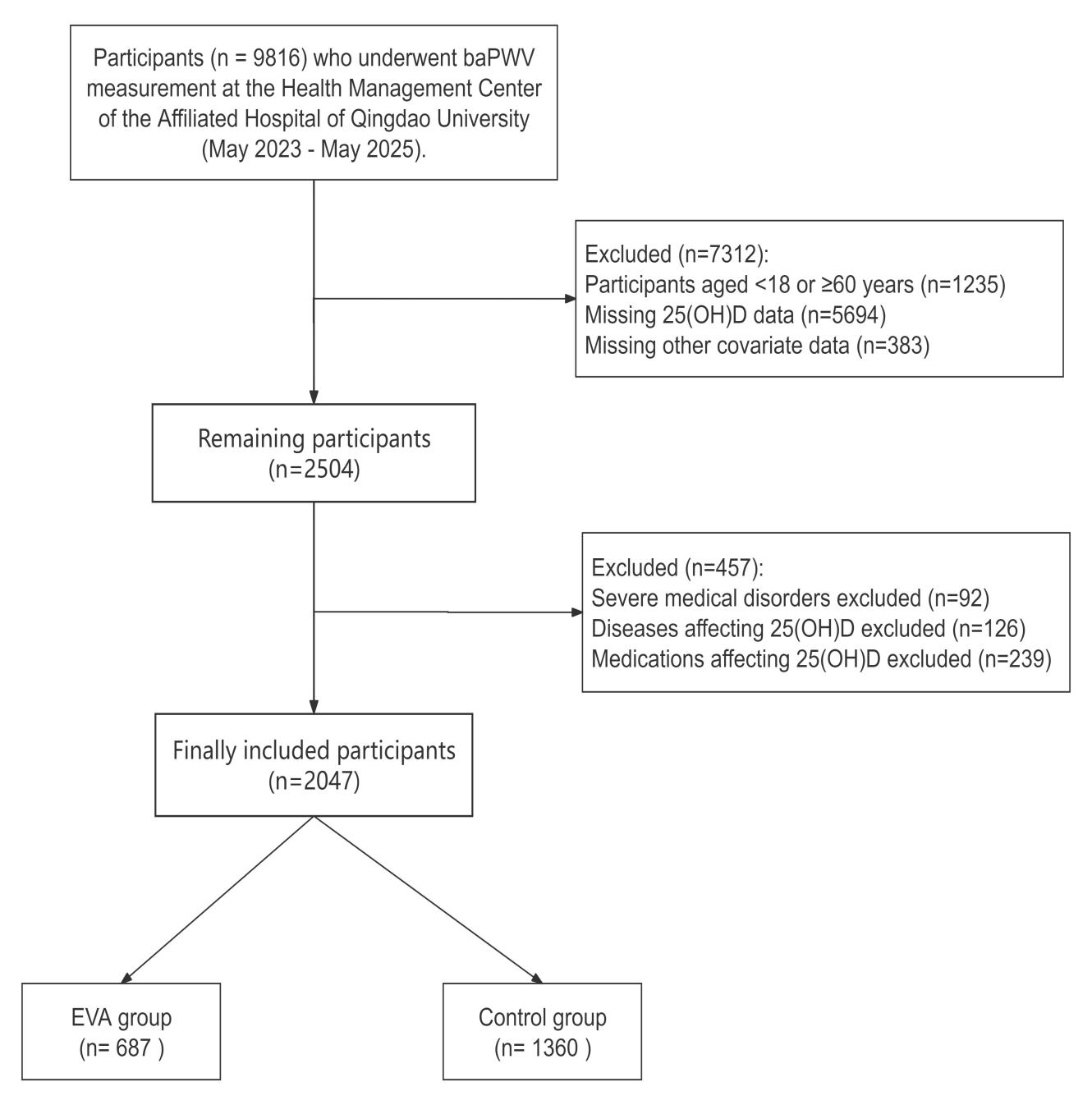


**S2 Fig. Reference curves of baPWV.** (A) Reference curve for baPWV in healthy males. As shown in the figure, the average value of bilateral baPWV in the study subject has exceeded 2 standard deviations (2SD) above the mean for the same age group, meeting the diagnostic criteria for early vascular aging (EVA). (B) Reference curve for baPWV in healthy females. As shown in the figure, the average value of bilateral baPWV in the study subject falls within the normal mean range, serving as the normal control group.

**
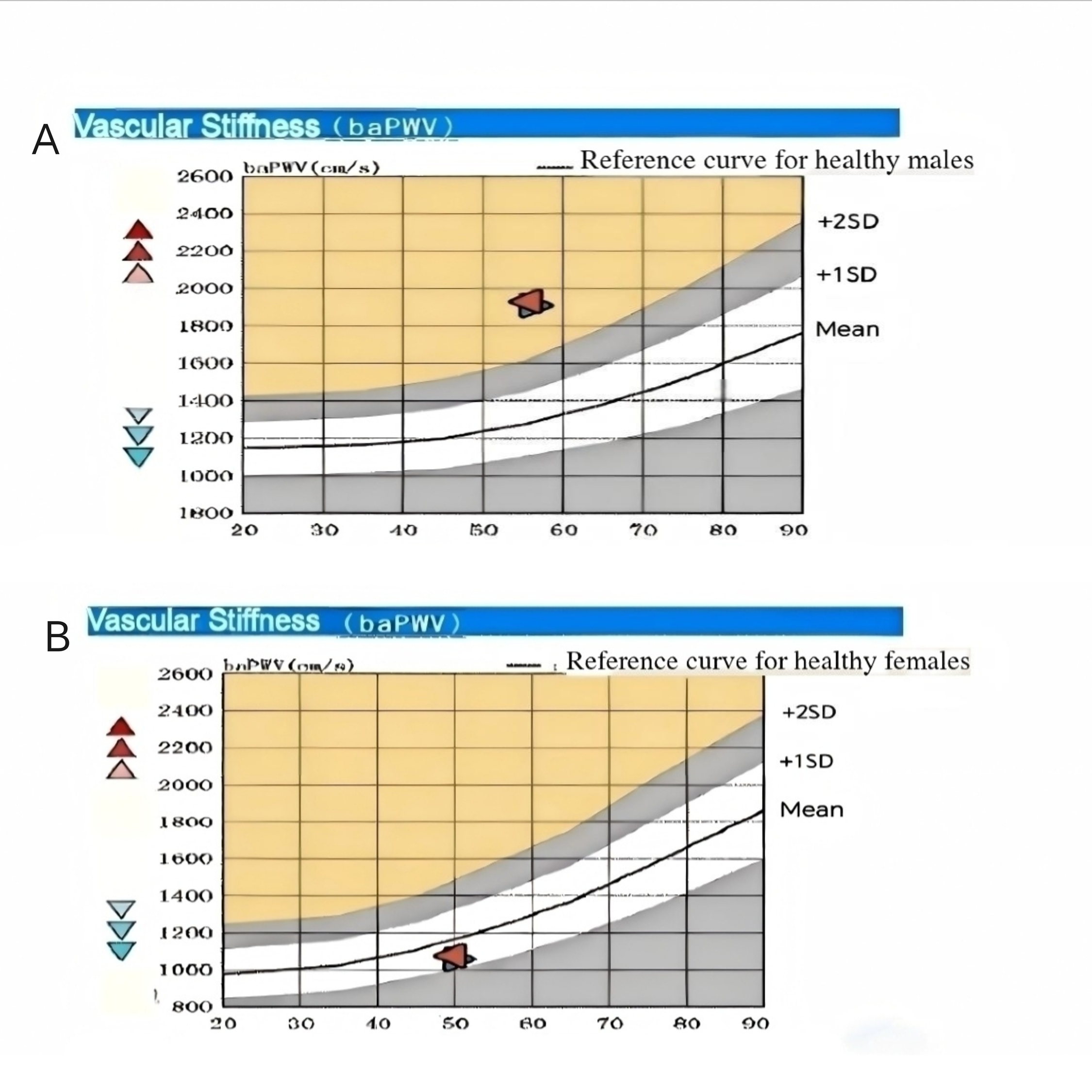
**

## Supplementary table

**S1 Table. The VIFs (Variance Inflation Factors, as measures of the multicollinearity) in the additional regression model 3**

| **Variables** | **VIFs** |
| --- | --- |
| Age (years) | 1.716 |
| Sex, n(%) | 2.289 |
| BMI( kg/m^2^) | 1.062 |
| Hypertension, n(%) | 1.121 |
| Diabetes, n(%) | 2.060 |
| Smoking status, n (%) | 1.333 |
| Alcohol drinking status , n(%) | 1.367 |
| Blood collection month, n(%) | 1.125 |
| 25(OH)D（ng/mL） | 1.205 |
| SBP (mmHg) | 2.655 |
| DBP(mmHg) | 2.817 |
| TG ( mmol/L) | 3.654 |
| TC ( mmol/L) | 22.839 |
| LDL-C ( mmol/L) | 17.974 |
| HDL-C ( mmol/L) | 4.000 |
| FBG (mmol/L) | 2.391 |
| HbA1c(%) | 2.116 |
| Cr (μmol/L) | 1.380 |
| UA (μmol/L) | 1.730 |
| Hcy (μmol/L) | 1.340 |

BMI, body mass index; SBP, Systolic Blood Pressure; DBP, Diastolic Blood Pressure; TG, triglyceride; TC, total cholesterol; LDL-C, low-density lipoprotein cholesterol; HDL-C, high-density lipoprotein cholesterol; FBG, fasting blood glucose; HbA1c, glycated hemoglobin; Cr, Creatinine; UA, uric acid; Hcy, Homocysteine
