# Supplementary material for: Association between serum 25-hydroxyvitamin D levels and Early Vascular Aging in young and middle-aged adults
Source: PLoS One. 2026 May 20;21(5):e0349409. doi: 10.1371/journal.pone.0349409 (PMC13189320; doi:10.1371/journal.pone.0349409)
Supplement: Attachment — Submitted filename: Response to Reviewers.doc [file pone.0349409.s004.doc]

Dear Dr. Jaalkhorol,

Thank you for the opportunity to revise our manuscript. We have carefully addressed all journal requirements:

1. Style requirements: We have ensured the manuscript fully adheres to PLOS ONE style requirements, including file naming conventions.

2. Ethics statement: The ethics statement now appears exclusively in the Methods section and has been removed from all other sections.

3. Data availability: We have uploaded the minimal anonymized data set as Supporting Information files (S1 Dataset) to ensure reproducibility while protecting participant privacy. There are no legal or ethical restrictions on data sharing; we chose this approach for simplicity. The Data Availability statement has been updated accordingly.

4. Citations: We have reviewed all reviewer recommendations regarding citations and incorporated relevant references where appropriate.

We have also carefully addressed the comments provided by the two reviewers, with our point-by-point responses detailed below. The specific line and page numbers refer to the Manuscript without tracked changes version, which are highlighted in green.

**Reviewer #1:**

*Comments1: The authors state that EVA is “a significant autonomous predictor of cardiovascular risk among individuals in their youth and middle age” (L52) and cite reference [3] in support of this statement. However, reference [3], as indicated by its title, addresses early vascular aging in children and adolescents, which does not correspond to the “youth and middle-aged” population studied here. It is unclear whether the current study population should be considered as having EVA according to this definition. Please clarify this issue and provide more appropriate references supporting the relevance of EVA in young and middle-aged adults.*

We sincerely thank you for your meticulous review. We fully acknowledge your valuable comment regarding the incorrect citation. Your observation is entirely accurate: the original reference [3] indeed focused exclusively on children and adolescents (<18 years), whereas our study population consists of adults aged 18-60 years. We sincerely apologize for this error.

We have corrected the references as follows: the inappropriate citation of reference [3] has been removed and replaced with references [3], [4], and [5], as detailed below:

[1] Bruno RM, et al. Early and Supernormal Vascular Aging: Clinical Characteristics and Association With Incident Cardiovascular Events. Hypertension . 2020 Nov;76(5):1616-1624.

[2] Cao Q, et al. Association of Early and Supernormal Vascular Aging categories with cardiovascular disease in the Chinese population. Front Cardiovasc Med . 2022 Aug 11;9:895792.

[3] Han M, et al. Early vascular aging determined by brachial-ankle pulse wave velocity and its impact on ischemic stroke outcome: a retrospective observational study. Sci Rep . 2024 Jun 13;14(1):13659.

All these newly added references investigated adult populations (including young and middle-aged adults), appropriately supporting the application of the EVA definition in our study cohort. We believe these revisions adequately address your concerns regarding the consistency between our study population and the EVA concept.

Thank you again for this important correction, which has significantly improved the rigor of our manuscript.

*Comments2: The authors state that “evidence regarding its relationship with EVA remains limited” (L59). However, the study population comprises adults aged ≥18 to <60 years, and numerous previous studies have examined the association between vitamin D status and cardiovascular disease or vascular function in middle-aged populations. Please cite appropriate literature and more clearly justify the novelty and significance of the present study.*

We sincerely appreciate the reviewer's valuable comments. We would like to clarify the novelty and significance of our study as follows:

We acknowledge that previous studies have extensively investigated the associations between vitamin D and cardiovascular disease (CVD) or atherosclerosis (AS) in middle-aged populations, as cited in references [11-13] (updated). However, early vascular aging (EVA) represents a distinctly different pathophysiological concept. First proposed by Nilsson in 2008, EVA specifically refers to the accelerated aging of biological vascular age relative to chronological age, emphasizing degenerative changes in vascular structure and function that occur prior to or independent of overt atherosclerotic plaque formation. While atherosclerosis focuses on lipid-driven plaque development, EVA concerns premature vascular aging, increased arterial stiffness, and endothelial dysfunction as an aging-related phenotype. Although AS and vascular aging may coexist, they differ in lesion location, risk factors, and pathogenetic mechanisms. Importantly, EVA can occur in arteries without AS, constituting a distinct aging phenotype. Consequently, evidence linking vitamin D to CVD/atherosclerosis cannot be directly extrapolated to EVA.

Furthermore, previous studies examining vitamin D and "arterial stiffness" have typically employed a uniform cutoff of PWV >1400 cm/s without accounting for age and sex, potentially misclassifying young individuals with pathologically stiffened vessels as "normal." The EVA concept addresses this limitation by adjusting for age and sex, enabling identification of high-risk individuals whose biological vascular age exceeds their chronological age—a population that traditional AS studies may overlook. The introduction of the EVA concept holds significant implications for primary prevention of CVD. To our knowledge, no study has specifically investigated the association between vitamin D and EVA defined by age- and sex-specific PWV thresholds.

This study presents several innovative findings. We are the first to reveal: (1) a non-linear relationship and threshold effects; (2) sex-specific differences, with a significant negative association observed in men but not in women, potentially reflecting the regulatory effects of sex hormones on vitamin D metabolism and vascular responses; and (3) BMI-specific effects, with significant associations observed in overweight/obese individuals, suggesting a potential role of vitamin D in obesity-related vascular aging.

We have revised the Introduction accordingly (see Lines 51-53 and 58-61 on page 3), explicitly distinguishing the established associations between vitamin D and atherosclerosis from the understudied specific relationship between vitamin D and EVA as an accelerated aging phenotype.

*Comments3:With regard to cardiovascular disease prevention by vitamin D supplementation, many intervention studies, including well-designed randomized controlled trials, have already been conducted. As the authors themselves note, “recent large-scale trials have not conclusively demonstrated cardiovascular benefits of vitamin D supplementation” (L61). In this context, the authors should more clearly articulate the scientific and clinical rationale for conducting the present cross-sectional study.*

We sincerely thank the reviewer for the valuable comment. You have raised a critical question regarding the scientific rationale for conducting this cross-sectional study in the context of existing RCT evidence. Indeed, while recent large-scale randomized controlled trials have failed to demonstrate cardiovascular benefits of vitamin D supplementation in the general population [14-17], the association between vitamin D deficiency and elevated cardiovascular risk persists in observational studies. This discrepancy underscores the significance of our study. The divergence between observational associations and interventional outcomes suggests that the relationship between vitamin D and vascular health may involve threshold effects or population heterogeneity—factors that broad supplementation trials cannot capture.

Importantly, although early vascular aging (EVA) addresses the limitations of traditional fixed diagnostic criteria through its individualized definition stratified by age and sex (>2SD), evidence regarding its specific relationship with vitamin D remains limited. This study does not aim to replicate efficacy trials of vitamin D supplementation; rather, it seeks to elucidate the mechanistic association between vitamin D and EVA as a specific vascular aging phenotype.We have supplemented the Introduction with cientific rationale to clarify the necessity of this study(see Lines 63-67 on page 4)

*Comments4: EVA is defined as baPWV exceeding the mean +2 standard deviations for individuals of the same age and sex. More detailed justification for this cutoff is required. In particular, the source of the reference values embedded in the Omron device should be clearly described, and relevant validation studies demonstrating the applicability of this definition in Chinese populations should be cited.*

We thank the reviewer for the thorough review. Regarding the source of the reference database, we provide the following clarifications: The reference curves utilized in this study were derived from the built-in standard database of the Omron BP-203RPE III device, which comprises data from approximately 12,000 Chinese healthy subjects. These reference populations were all screened through health examinations, with no history of arteriosclerotic diseases, absence of arteriosclerotic risk factors such as hypertension, diabetes, and dyslipidemia, and all had blood pressure within normal ranges (systolic blood pressure <140 mmHg, diastolic blood pressure <90 mmHg). This large-scale cohort encompasses various age groups and genders, with statistical corrections and stratification applied. Furthermore, the subjects in our study are of the same ethnicity (Chinese adults) and meet the characteristics of the aforementioned reference population; therefore, the adoption of these reference curves is scientifically justified.

These data originate from the technical specification documents and device manuals provided by Omron Corporation. This represents a standard approach for presenting performance parameters of medical devices, consistent with conventional reporting standards for clinical equipment research. As this database constitutes an internal standard established by the device manufacturer, its detailed demographic data have not been published as independent papers; however, it has been validated through multiple subsequent independent studies. A prospective study by Cao et al. (2022), published in Frontiers in Cardiovascular Medicine , demonstrated that early vascular aging (EVA) defined using this device's reference criteria was significantly associated with cardiovascular disease risk in Chinese populations. Additionally, research by Sang et al. (2020), published in Medical Science Monitor , validated the applicability of this device (BP-203RPE III) in healthy elderly Chinese populations. The Chinese user manual and manufacturer technical support documentation for the Omron BP-203RPE III device used in this study also explicitly indicate these reference intervals for vascular physiological age assessment in healthy Chinese adults, demonstrating good reference value.

We have detailed in the Methods section that the baPWV reference curves were derived from the device’s built-in database(see Lines 104-110 on page 6) (established from a large health-check population of Chinese healthy adults and stratified by age and sex; see Supplementary Figure S2) and added references [4,18] to validate its applicability in Chinese populations.

*Comments5:Because this is a cross-sectional study, causal relationships between serum 25(OH)D and EVA cannot be inferred. In the Discussion and Conclusion, causal wording (e.g., “protective effect,” “benefits”) should be replaced with more cautious terms such as “association” or “correlation.”*

Thank you very much for your insightful comments and careful examination of our manuscript. We sincerely appreciate your valuable suggestion regarding the cautious interpretation of our findings.

We fully acknowledge that as a cross-sectional study, causal relationships between serum 25(OH)D and EVA cannot be established. We apologize for any causal wording that appeared in the original manuscript. Following your suggestion, we have carefully revised the Discussion section (see Lines 39、284-286、323-329) and the Conclusion section to replace all causal terms such as "protective effect" and "benefits" with more cautious expressions including "association," "inverse association," and "correlation."

The revised manuscript now clearly reflects the observational nature of our study and avoids any implication of causality between vitamin D status and EVA.

*Comments6:The method used to select study participants should be described more clearly. In addition, the authors should state whether a priori sample size estimation was performed and provide the rationale for the sample size.*

Thank you for your valuable suggestion. We have revised the Methods section to clearly describe the participant selection process(see Lines 74-86 on page 4) . Specifically, we now state that participants were initially screened from 9,816 adults who underwent baPWV measurement, and detail the sequential exclusion criteria (age <18 or ≥60 years, missing data, severe medical disorders, conditions affecting 25(OH)D levels, pregnancy/lactation, and recent medication use). The complete flowchart of participant inclusion is illustrated in Supplementary Figure 1 (FS1 Fig) for full transparency.

Regarding the sample size estimation: As this study was a cross-sectional study utilizing existing health examination data (retrospectively collected from May 2023 to May 2025), a formal a priori sample size calculation was not performed. Instead, we conducted a post-hoc power analysis to verify statistical adequacy. Using G*Power 3.1.9.7 (z-test for logistic regression, Demidenko method), based on the observed effect size (OR = 0.81), event rate (33.6%, 687/2,047), α = 0.05 (two-sided), and total sample size (n = 2,047), the calculated statistical power was >99%, far exceeding the conventional 80% threshold (see figure below).

Furthermore, according to the EPV (Events Per Variable) principle, our fully adjusted model included 687 outcome events and approximately 18 covariates, yielding an EPV ratio of 38:1, significantly higher than the recommended minimum standard of 10:1. Therefore, both the post-hoc power analysis and EPV assessment confirmed that our sample size (n = 2,047) was fully adequate to reliably detect the association between 25(OH)D and EVA

*Comments7:The Methods section should specify how the threshold value of 17.9 ng/mL was determined, including the algorithm or statistical procedure used.*

We appreciate your valuable comment regarding the clarification of the threshold determination method. We have supplemented and refined the description in the Statistical Analysis section to detail the specific algorithm and statistical procedure used to determine the threshold value of 17.9 ng/mL(see Lines 145-152 on page 7).

Specifically, the threshold was determined using segmented regression analysis (piecewise linear regression) with the segmented package in R. Following the detection of a significant non-linear association by restricted cubic spline (RCS) analysis (P for non-linearity = 0.015), a two-piecewise linear logistic regression model was fitted. The threshold was not pre-specified a priori ; rather, it was estimated from the data under a one-breakpoint model (npsi = 1) using an iterative algorithm. The initial value of the breakpoint parameter (psi) was set to the median of serum 25(OH)D concentrations. The optimal threshold of 17.9 ng/mL was selected based on the minimization of the Akaike Information Criterion (AIC) and Bayesian Information Criterion (BIC), with consideration of the standard errors of model parameters to ensure estimation precision.

*Comments8:The authors present ROC curves and AUC values to support the “clinical utility” of the threshold. However, the validity of diagnostic performance evaluation based on cross-sectional data is questionable. The purpose of the ROC analysis should be clarified.*

Thank you for raising this important methodological concern. We agree that using ROC curves to evaluate "diagnostic performance" is inappropriate for cross-sectional data, as this study design precludes the assessment of diagnostic accuracy. Upon reflection, the ROC analysis was initially added to demonstrate the discriminative ability of the threshold for risk stratification. However, as correctly pointed out, this may misleadingly imply a diagnostic utility that our cross-sectional design cannot support. We have therefore removed the ROC analysis and associated AUC values from the manuscript.

*Comments9:In Table 2 and Figure 1, the reported odds ratios (e.g., OR = 0.98) appear to represent the change per 1 ng/mL increase in 25(OH)D. This should be explicitly stated in footnotes. Because a 1 ng/mL change may have limited clinical relevance, the authors are encouraged to additionally present estimated ORs per 10 ng/mL increase.*

Thank you for your valuable suggestion regarding the clinical relevance of the effect size presentation. We have carefully addressed your concerns as follows:

Table 2: Modified to per 10 ng/mL as recommended We have re-run the multivariate logistic regression models by dividing the continuous 25(OH)D variable by 10. The revised results now present ORs per 10 ng/mL increase (Model 3: OR = 0.81, 95% CI: 0.70–0.94, P = 0.008), indicating a 19% reduction in EVA risk per 10 ng/mL increment. This presentation indeed enhances clinical interpretability compared to the previous per 1 ng/mL metric. The footnote has been updated to explicitly state: "ORs for continuous 25(OH)D represent the change per 10 ng/mL increase."

Figure 1 (RCS curve): Retained per 1 ng/mL for methodological rigor We respectfully retained the original per 1 ng/mL scale for the restricted cubic spline (RCS) analysis for the following reasons:

The RCS curve aims to identify the non-linear relationship and the precise inflection point at 17.90 ng/mL. A 10 ng/mL interval would oversmooth the curve and obscure the detailed turning pattern around this clinically significant threshold. Below the threshold (<17.90 ng/mL), converting OR = 0.90 (per 1 ng/mL) to per 10 ng/mL would yield OR ≈ 0.35, suggesting a 65% risk reduction. Such an extreme effect size might exceed conventional clinical expectations for vitamin D supplementation and potentially raise questions regarding biological plausibility.Above the threshold (>17.90 ng/mL), the association is minimal (OR = 0.98 per 1 ng/mL; OR ≈ 0.82 per 10 ng/mL). The per 1 ng/mL scale better captures the attenuation of effect in the sufficient range.

Explicit footnotes for clarity across all figures To prevent any ambiguity regarding units, we have added standardized footnotes:Table 2: "ORs for continuous 25(OH)D represent the change per 10 ng/mL increase." Figure 1: "Odds ratios (ORs) represent the change per 1 ng/mL increase in serum 25(OH)D." Figure 2: "Odds ratios (ORs) represent the change per 1 ng/mL increase in serum 25(OH)D."

This approach ensures clinical relevance in the main association table while preserving the methodological integrity of the dose-response curve and subgroup analyses for precise threshold identification and stratified effect estimation. We believe this addresses your concerns while maintaining scientific rigor across all analyses.

*Comments10:Regarding the finding that the association was significant only in men, potential explanations beyond hormonal status, fat distribution, and lifestyle factors—such as differences in sample size between men and women—should also be discussed.*

Thank you for your insightful comment regarding the sex-specific findings in our study. We appreciate your suggestion to consider methodological factors alongside biological explanations.

We have revised the Discussion section(see Lines 300-304 on page 16) to address this point. This modification acknowledges that sample size differences between sexes may have contributed to the observed association while maintaining our discussion of biological mechanisms. We believe this provides a more balanced interpretation of our findings.

*Comments11:If the authors suggest potential implications for vitamin D supplementation, they should carefully consider and acknowledge that randomized controlled trials have not demonstrated clear cardiovascular benefits.*

Thank you for this important comment. We fully agree that our cross-sectional study cannot establish causality or imply therapeutic benefits of vitamin D supplementation.

Following your suggestion, we have carefully removed the sentence "It provides a basis for further investigation into the impact of serum 25(OH)D concentrations on the intervention of EVA" from the Discussion section, as it inappropriately suggested an intervention potential that cannot be inferred from observational data.

We acknowledge that existing randomized controlled trials have not demonstrated significant cardiovascular benefits from vitamin D supplementation. Our study only identifies an association between lower 25(OH)D levels and EVA, which may reflect confounding factors or reverse causation rather than a modifiable causal relationship. We have revised the Discussion to emphasize that our findings provide observational clues only and should not be interpreted as evidence supporting vitamin D supplementation for cardiovascular prevention.

*Comments12: In Table 1, “Famale” should be corrected to “Female.”*

Thank you for pointing out this typographical error. We have corrected "Famale" to "Female" in Table 1.

**Reviewer #2:**

*Comments1:Would you explain why participants from different seasons were essential?*

Thank you for this important question. The continuous enrollment strategy implemented between May 2023 and May 2025 inherently captured participants from different seasons, a feature that proved essential for the following reasons:

Serum 25(OH)D levels exhibit well-established seasonal fluctuations, typically peaking in summer (May–October) and declining in winter (November–April) due to variations in ultraviolet B (UVB) exposure. Given the substantial nature of this biological variation, failure to adjust for it could confound the association between 25(OH)D and EVA. Specifically, if participants with EVA were disproportionately enrolled during seasons with lower vitamin D levels, the observed association might reflect seasonal variation rather than a true pathophysiological relationship. To address this, we included "blood collection month" (dichotomized as May–October versus November–April) as a covariate in Model 2 and Model 3, thereby statistically adjusting for this potential confounder. This adjustment ensures that the observed association between 25(OH)D and EVA is independent of seasonal effects and allows the findings to be generalizable across different periods of the year, rather than being limited to a specific season.

In summary, the continuous enrollment strategy naturally ensured coverage of participants across different seasons, which was essential for two reasons: first, it enabled us to statistically adjust for seasonal variations in 25(OH)D levels, thereby isolating the true association with EVA; second, it enhances the generalizability of our findings to year-round clinical practice, rather than limiting them to a specific season.

*Comments2：The sample size looks enough, but do you have any assumptions that Q1 was equal to Q3, while Q4 is the highest?*

Thank you for this important observation and for giving us the opportunity to clarify our methodology. We apologize if our presentation caused any confusion regarding the group comparisons.

To address your question directly: we did not assume that Q1 equals Q3. We would like to explain our analytical approach and the interpretation of the results in detail:

1）Regarding the Sample Distribution The Q1-Q4 groups represent quartiles based on serum 25(OH)D levels, with each group theoretically comprising 25% of the total sample (n=2,047). The similar sample sizes between Q1 (25.55%, n=523) and Q3 (25.16%, n=515) simply reflect the natural mathematical distribution of quartile-based categorization, rather than any statistical assumption or hypothesis that Q1 equals Q3.

2）Regarding the Reference Group and Comparison Strategy In our logistic regression analysis, Q1 (the lowest 25(OH)D quartile, <13.6 ng/mL) served as the sole reference group. Q2, Q3, and Q4 were each independently compared against Q1, with no presumption of equal effects between any groups, including non-adjacent quartiles.

3）Interpretation of the Findings: Threshold Effect We observed that Q2 and Q3 did not differ significantly from Q1, while only Q4 (the highest quartile, ≥23.7 ng/mL) showed a statistically significant protective effect (Model 3: OR=0.58, 95%CI 0.42-0.80, p<0.001). This pattern suggests a potential threshold effect, where a significant reduction in EVA risk may only occur when vitamin D levels reach the highest quartile, rather than following a simple linear gradient.

4）Regarding Q4 as the Highest Group Q4 was defined as the highest quartile based on the actual distribution of 25(OH)D concentrations in our sample (≥23.7 ng/mL). Comparing the highest (Q4) and lowest (Q1) quartiles is standard practice in such analyses to evaluate the effect across the full exposure range.

We hope this clarification addresses your concern. We did not assume equality between any quartiles; rather, the analysis was designed to test for independent associations while respecting the ordinal nature of the exposure variable.

*Comments3：Can you explain the result of HbA1c? It was 5.70 in the total group, but 5.70 in the control and 5.75 in the EVA group. Why is the total group the same as the control group?*

Thank you for your careful observation regarding the HbA1c results. We appreciate the opportunity to clarify this finding. The original data were re-checked, and the above values accurately reflect the dataset used. The identical medians for the overall group and control group (5.70%) are mainly attributable to the imbalance in group sample sizes and the characteristics of the data distribution. Specifically, the control group included 1,360 participants (66.4% of the total), whereas the EVA group included 687 participants (33.6%); consequently, the overall median is heavily weighted toward and coincides with the control group distribution at the 50th percentile. However, the interquartile ranges indicated meaningful differences between the two groups: the control group showed a relatively narrower distribution (5.40%–5.80%), whereas the EVA group exhibited greater dispersion (5.50%–6.00%), suggesting higher variability in glycemic fluctuations in the EVA cohort. Furthermore, despite the identical medians in the overall and control groups, the Mann–Whitney U test detected a statistically significant difference between the two groups (P < 0.001), driven primarily by disparities in the upper quartiles rather than by the medians alone.

*Comments4：In line 177, I could not understand the "three models". Please write that they are Q1, Q2, and Q3.*

Thank you for pointing out this ambiguity. We sincerely apologize for the confusion. The "three models" mentioned in the text refer to three multivariate logistic regression models with varying degrees of covariate adjustment (Model 1: unadjusted; Model 2: adjusted for age, sex, blood collection month, BMI, smoking status, drinking status, hypertension, and diabetes; Model 3: further adjusted for SBP, DBP, TG, HDL-C, FBG, UA, Cr, HbA1c, and Hcy on the basis of Model 2), as presented in Table 2. The quartiles (Q1, Q2, Q3, Q4) represent the categorical groupings of serum 25(OH)D levels (see "Assessment of serum 25(OH)D levels" in the Methods section for details). We have revised the text to explicitly state(see Lines 188-191 on page 11). This clarification should make it clear that "models" refers to the regression models, not the quartile groups.

*Comments5：In line 178, EVAwere was type error.*

Thank you for catching this typographical error. We have corrected "EVAwere" to "EVA were" in the revised manuscript(see Lines 189 on page 11).

*Comments6：In line 213, drinking status is a little bit confusing. Please clarify whether it is alcohol drinking or a water intake habit.*

Thank you for identifying this ambiguity. To clarify, we have revised "drinking status" to "alcohol drinking status" in Line 213. We have also updated all similar expressions throughout the manuscript (including footnotes in Table 1 and Table 2, and Figure 2) to ensure terminological consistency. These revisions confirm that the term specifically refers to alcohol consumption habits rather than water intake.

*Comments7：In the discussion (line 243), the "older age groups" statement is unclear. Please clarify whether it is above 60 or others.*

Thank you for identifying this ambiguity. We apologize for the imprecise terminology. The phrase "older age groups" was indeed misleading. Our analysis actually showed that the EVA group had a significantly older mean age compared to the control group (as a baseline characteristic difference), rather than referring to specific age categories (e.g., >60 years). To accurately reflect this group difference in average age, we have revised the statement to clarify that EVA was associated with older average age in the affected group(see Lines 254 on page 14).This eliminates the confusion regarding age group definitions.

Once again, we sincerely thank the reviewers for their careful consideration and valuable comments on our manuscript. We have carefully addressed each point and revised the manuscript accordingly, with the aim of further improving the scientific rigor and clarity of our work. We respectfully ask that you consider our manuscript favorably and grant us the opportunity to be published in your journal.

Sincerely,

Rui Hu

The Affiliated Hospital of Qingdao University

March 22, 2026
