# Supplementary material for: Association between serum 25-hydroxyvitamin D levels and Early Vascular Aging in young and middle-aged adults
Source: PLoS One. 2026 May 20;21(5):e0349409. doi: 10.1371/journal.pone.0349409 (PMC13189320; doi:10.1371/journal.pone.0349409)
Supplement: Attachment — Submitted filename: Response_to_Reviewers_auresp_2.doc [file pone.0349409.s005.doc]

**Dear Dr. Jaalkhorol**

We sincerely thank PLOS ONE for providing us with the opportunity to revise our manuscript. We are deeply grateful to the Academic Editor and both reviewers for their constructive comments and thorough evaluation, which have substantially enhanced the quality of our work. Below are our point-by-point responses to the reviewers' comments:

**Reviewer #1:** *The authors used the built-in reference values of the Omron device to define EVA. They state that these reference values were derived from approximately 12,000 healthy Chinese subjects. However, detailed information about the reference population is limited and appears to be obtained from technical specifications and device manuals rather than from published scientific articles. Consequently, neither the readers nor the authors can determine from which population the reference subjects were drawn, who selected them, how they were selected, how health information was obtained, or how reliable the information was.

In addition, the authors added references #4 and #18 as supporting evidence for the use of these reference values. However, these studies report the validity of baPWV in predicting cardiovascular disease risk and do not demonstrate that the built-in reference values represent valid normal reference values for the Chinese population. Therefore, this issue represents an important limitation of the present study and should be clearly acknowledged in the Limitations section.*

We sincerely appreciate the reviewer's careful and constructive feedback on this critical methodological issue. The reviewer is absolutely correct, and we fully acknowledge this important limitation.

Revisions made:

To transparently address this concern, we have made the following specific modifications:

1. In the Methods section (Assessment of EVA using baPWV): We have removed the previous statement attempting to justify the reference curves using references [4] and [18] (previously located at lines 109-112 on page 6).
2. In the Discussion section (Discussion, lines 318-325 on page 17): We have added relevant content to explicitly acknowledge this limitation:"Although the predictive validity of baPWV measurement for cardiovascular risk has been well established [4,18], the validity of the built-in reference thresholds as normal values for the Chinese population has not been specifically validated in independent studies, as detailed demographic characteristics of the source population for these reference curves are only available in device technical specifications and manuals, lacking direct support from peer-reviewed scientific literature." We agree with the reviewer that this represents an important limitation of our study, as neither readers nor the authors can fully ascertain the selection criteria, geographic origin, or data reliability of the reference population. While we maintain that the use of these curves is reasonable given the ethnic consistency between our cohort and the reference population (as stated in the manufacturer's documentation), we now explicitly recognize that the lack of peer-reviewed validation for the reference thresholds .

Thank you for this valuable guidance in improving the scientific rigor of our manuscript.

All revisions have been highlighted in the "Revised Manuscript with Track Changes" file. We believe these revisions provide appropriate transparency regarding the methodological limitations and will assist readers in interpreting our findings with due caution.

We also extend our gratitude to Reviewer #2 for their positive comments and encouragement regarding the potential impact of our study. We hope these revisions satisfactorily address the reviewers' concerns and meet PLOS ONE's publication criteria.

Sincerely,

Rui Hu The Affiliated Hospital of Qingdao University
